# Cochlear implantation in autistic children with profound sensorineural hearing loss^☆^

**DOI:** 10.1016/j.bjorl.2016.10.012

**Published:** 2016-11-19

**Authors:** Magdalena Lachowska, Agnieszka Pastuszka, Zuzanna Łukaszewicz-Moszyńska, Lidia Mikołajewska, Kazimierz Niemczyk

**Affiliations:** Medical University of Warsaw, Hearing Implants Center, Department of Otolaryngology, Warsaw, Poland

**Keywords:** Austism, Hearing loss, Hearing aid, Speech perception, Cochlear implant, Autismo, Perda de audição, Aparelho auditivo, Percepção da fala, Implante coclear

## Abstract

**Introduction:**

Cochlear implants have become the method of choice for the treatment of severe-to-profound hearing loss in both children and adults. Its benefits are well documented in the pediatric and adult population. Also deaf children with additional needs, including autism, have been covered by this treatment.

**Objective:**

The aim of this study was to assess the benefits from cochlear implantation in deafened children with autism as the only additional disability.

**Methods:**

This study analyzes data of six children. The follow-up time was at least 43 months. The following data were analyzed: medical history, reaction to music and sound, Ling's six sounds test, onomatopoeic word test, reaction to spoken child's name, response to requests, questionnaire given to parents, sound processor fitting sessions and data.

**Results:**

After cochlear implantation each child presented other communication skills. In some children, the symptoms of speech understanding were observed. No increased hyperactivity associated with daily use cochlear implant was observed. The study showed that in autistic children the perception is very important for a child's sense of security and makes contact with parents easier.

**Conclusion:**

Our study showed that oral communication is not likely to be a realistic goal in children with cochlear implants and autism. The implantation results showed benefits that varied among those children. The traditional methods of evaluating the results of cochlear implantation in children with autism are usually insufficient to fully assess the functional benefits. These benefits should be assessed in a more comprehensive manner taking into account the limitations of communication resulting from the essence of autism. It is important that we share knowledge about these complex children with cochlear implants.

## Resumo

**Introdução:**

Os implantes Cocleares tornaram-se o método de escolha para o tratamento da perda auditiva severa a profunda em crianças e adultos. Seus benefícios estão bem documentados nas populações pediátrica e adulta. Também as crianças surdas com necessidades adicionais, incluindo autismo, têm sido incluídas nesse tratamento.

**Objetivo:**

O objetivo desse estudo foi avaliar os benefícios do implante coclear em crianças surdas com autismo como única deficiência adicional.

**Método:**

Esse estudo analisa os dados de seis crianças. O tempo de seguimento foi de pelo menos 43 meses. Foram analisados os seguintes dados: histórico médico, a reação à música e ao som, teste dos seis sons de Ling, teste de palavra onomatopaica, reação ao nome falado da criança, resposta a pedidos, questionário aplicado aos pais, sessões de ajustes e dados do processador de som.

**Resultados:**

Após o implante coclear, cada criança apresentou outras habilidades de comunicação. Em algumas crianças, foram observados sinais de compreensão da fala. Não foi observado aumento de hiperatividade associada com o uso diário de implante coclear. O estudo mostrou que em crianças autistas a percepção é muito importante para a sensação de segurança da criança e torna o contato com os pais mais fácil.

**Conclusão:**

Nosso estudo mostrou que a comunicação oral não é uma meta realística provável em crianças com implantes cocleares e autismo. Os resultados do implante mostraram benefícios variáveis entre as crianças. Os métodos tradicionais de avaliação dos resultados do implante coclear em crianças com autismo são geralmente insuficientes para avaliar plenamente os benefícios funcionais. Esses benefícios devem ser avaliados de forma mais abrangente, tendo em conta as limitações de comunicação resultantes da essência do autismo. É importante que compartilhemos conhecimentos sobre essas complexas crianças com implantes cocleares.

## Introduction

Cochlear implants have become the method of choice for the treatment of severe-to-profound hearing loss in both children and adults. Its benefits are well documented in the pediatric and adult population. Also deaf children with additional needs, including autism, have been covered by this treatment. However, there is limited available literature regarding benefits of cochlear implantation in children with autism as the only additional disability.^1^, ^2^, ^3^, ^4^, ^5^, ^6^

More and more children with multiply handicaps have been receiving cochlear implants in our department as well,^7^ among them 6 were diagnosed with autistic disorder as the only additional disability next to deafness and their results have been assessed and discussed in this study.

Etiology of autism still remains unknown. The disorder description covers impaired social interaction, abnormal verbal and non-verbal communication, restricted repetitive and stereotyped patterns of behavior. The symptoms usually develop in early childhood, mainly in the first two-three years of life.^8^, ^9^ When it comes to autism and hearing, the data from evoked potential studies indicates that in autistic patients there is an abnormal cognitive processing of auditory information despite normal basic sensory perception.^10^, ^11^, ^12^, ^13^, ^14^, ^15^, ^16^ This is one of the reasons why cochlear implanted autistic children do not usually develop language skills as those with no additional disability.^1^, ^5^

The aim of this study was to assess the benefits from cochlear implantation in deafened children with autism as the only additional disability.

## Methods

This retrospective study analyzes data of 6 children with prelingual profound bilateral hearing loss and autistic disorder. Before the surgery all patients underwent very careful multidisciplinary evaluation to determine candidacy for cochlear implantation: severe to profound bilateral sensorineural hearing loss, prelingual onset of hearing loss, no benefit from appropriately fitted hearing aids, no medical nor radiological contraindications, desire in a family to improve child's hearing and communication with a child, good motivation running in the family. None of the presented children had completed and confirmed diagnosis of autism at the time of implantation (autism was suspected but the diagnosis was not clearly established at that time). Only one child seemed very clearly to be autistic before the implantation (patient no. 5, the oldest at the time of implantation).

All children underwent the implantation in our department. There were neither perisurgical nor postsurgical complications. All children were implanted unilaterally to the right ear with a multichannel cochlear implant. Five were implanted with Cochlear Nucleus, and one with Advanced Bionics (Table 1). Speech processor was activated one month after the surgery in all cases. All patients have been coming back to the department for regular follow-up and fitting sessions. All of them have been under multidisciplinary rehabilitation.

**Table 1:** Demographic information about implanted autistic patients.

Patient No.	Cause of deafness (present risk factors)	Age at implantation	Type of implant	Implanted ear (side)	NRT
1	Dandy-Walker syndrome (DWS), prematurity (32 Hbd), low birth weight (1930 g), gentamicin	21 months	Nucleus Cochlear	R	Yes
2	Prematurity (25 Hbd), low birth weight (740 g)	21 months	Nucleus Cochlear	R	Yes
3	Unknown	21 months	Nucleus Cochlear	R	Yes
4	Unknown	15 months	Advanced Bionics	R	Yes
5	Congenital Cytomegalovirus Infection (CMV)	29 months	Nucleus Cochlear	R	Yes
6	Prematurity (28 Hbd), low birth weight (960), Toxocariasis	26 months	Nucleus Cochlear	R	Yes

At the time of this study, the follow-up time was at least 43 months. The following data were analyzed: medical history, reaction to music and sound, Ling's six sounds test, onomatopoeic word test, reaction to spoken child's name, response to requests, questionnaire given to parents, sound processor fitting sessions and data.

The questionnaire given to parents consisted of 6 questions as listed below:

1. Does your child willingly use the sound processor?
2. How many hours a day the sound processor is on?
3. Does the child respond to his/her name in quiet with auditory cues only (no visual cues)?
4. Does the child spontaneously alert to environmental sounds?
5. Is the child's behavior affected while wearing his/her sound processor?
6. Does the family interactions with the child and within the family benefited from implant?

The impact that cochlear implantation has on autistic children is further illustrated by each patient's results and their family's experience.

The presented study conforms to The Code of Ethics of the World Medical Association (Declaration of Helsinki). This is a retrospective study and no free informed consent form for this study was needed, subject's identity was not divulged.

## Results

All six analyzed patients were males. The mean age at the time of cochlear implantation was 1.8 years old (SD = 0.3, max = 2.1, min = 1.4). The mean post-implantation follow-up time was 5.9 years (SD = 2.0, max = 8.5, min = 3.6).

The data revealed that in all children language development after implantation was very delayed both receptive and expressive language (Table 2). Every child represented different skills but none of them used gestures as a communication system. Only three children were turning head from side to side as “no”, and nodding the head as “yes”. When it comes to vocalization it was mainly screaming and no willingness to follow therapist's vocalizations but one child, the oldest ones, used a few spoken short words to communicate but only when he felt like doing so. This child and another one also responded to spoken requests – they did what they were asked for. All of the children presented poor eye contact.

**Table 2:** Individual reaction to sounds and spoken language in six autistic children with cochlear implants.

Patient No.	Reaction to music	Ling's 6 sounds test	Reaction to spoken name	Response to spoken requests
1	No	No	No	No
2	Yes (only to drum)	No	No	No
3	Yes (only to flute and drum)	No	No	No
4	Yes	Yes	Yes	Yes
5	Yes (only to drum)	No	No	No
6	Yes	Yes	Yes	Yes

The questionnaire results (Table 3) revealed that all children liked wearing sound processor for all day long every day. Half of the children responded to their names when their parents were calling them, and the same three children responded to environmental sound at home and known surroundings. According to the parents’ statements, most of the children presented reduced anxiety when wearing the sound processor. In two cases the behavior did not change despite the processor on but at the same time no increased hyperactivity associated with daily use cochlear implant was observed. All the families reported benefits in child's personal interaction with the family members and within the family after cochlear implantation.

**Table 3:** The questionnaire results. The parents responded to the following questions: (1) Does your child willingly uses the sound processor?; (2) How many hours a day the sound processor is on?; (3) Does the child respond to his/her name in quiet with auditory cues only (no visual cues)?; (4) Does the child spontaneously alert to environmental sounds?; (5) Is the child's behavior affected while wearing his/her sound processor?; and (6) Does the family interactions with the child and within the family benefited from implant? (The same order of questions is kept in the table).

Patient No.	Does he like wearing sound processor?	How many hours a day?	Response to name	Response to environmental sounds	Behavior changes	Better family interactions?
1	Yes	All day	No	No	None	Yes
2	Yes	All day	No	No	Reduced anxiety	Yes
3	Yes	All day	Yes	Yes	Reduced anxiety	Yes
4	Yes	All day	Yes	Yes	Reduced anxiety	Yes
5	Yes	All day	No	No	None	Yes
6	Yes	All day	Yes	Yes	Reduced anxiety	Yes

Data sound processor fitting sessions revealed that all children were mostly hyperactive during sessions. However, in four cases the child's cooperation during sessions was good, with one child needed to be fitted by only one person from our team. Only two children presented week cooperation. Four children allowed for Neural Response Telemetry (NRT) and impedance measurements to be fully performed without interruptions. However, the children's willingness to undergo those tests changed from session to session, that is why fitting sessions were mainly based on behavioral responses. One child, the oldest in the group, was able to perform free field audiometry. The caution had to be undertaken when fitting sound processor due to high sensitivity to sound in every child with autism, as observed in our group and in literature.^12^

## Discussion

The cochlear implants provide access to sound for severely to profoundly hearing impaired individuals, both children and adults, and they are widely accepted and effective treatment method for those patients. However, cochlear implantation in the autistic children remains a point of discussion and is supported by very limited literature so far, showing that they would not perform as well as their implanted peers who present no additional disabilities.^1^, ^2^, ^4^, ^5^, ^6^ It is known that autism severely interferes with language or learning process. Those children are likely to be dependent from their families or caregivers throughout their lives. For them, the usual goal of spoken language after cochlear implantation may be unrealistic. They pose a great challenge for implant teams and for those working with them and their families throughout the whole postimplant rehabilitation process.^1^, ^2^, ^4^ As shown in this study, in addition to those children's lack of ability to provide feedback about what they really hear, high sensitivity to even small sound changes makes programming and the assessment of functional gains in autistic children more difficult. Due to high sensitivity to acoustic stimuli the caution must be undertaken when fitting sound processor.^12^, ^17^

The benefits in implanted autistic children are different among them.^1^, ^5^ The evidence reveals that important outcomes should not be assessed by traditional outcome measures of speech perception and production.^1^ The assessment is usually more complex and time-consuming than in children with deafness as the only disability, and these should be considered on an individual basis. It is likely that type of assessments is non-standardized or informally developed or adapted. In our department, we make use of the following: observation through play, parental knowledge of their child's communication skills observed in everyday life, observation of routine situations in child's environment, questionnaire given to parents. In addition, during fitting sessions a child is given longer time to respond. Spoken perfection tests could not be applied because of the autism. We believe that families are excellent source of information^18^ regarding everyday behavior of their child and cochlear implantation benefits.

Based on our experience and those from other studies, receiving an implant by autistic children in most cases does not lead to the development of speech and language even after many years.^1^, ^5^ In their cases, communication skills should be viewed from a wider perspective. However we found out that sometimes children are much more capable than had been previously supposed and cochlear implantation enhance the autistic child's quality of life even when the language development itself benefits very little. In our study, only one child uses spoken just a few simple words to communicate which is very rare in implanted autistic children as shown in limited literature on this subject.^1^, ^5^ In our study, before the implantation parents’ expectations were higher and counseling was very important to prepare the parents for more realistic results. These expectations changed over time after cochlear implantation and fully established diagnosis of autism, they became more realistic given autism in their children, and again counseling was very helpful.

More common benefits from implantation in this group is response to a name, response to environmental sounds, reduced anxiety and better personal interaction with the family members. Those benefits were also reported by parents in our study; however, they vary among patients.^17^ In our study, after cochlear implantation each child presented different communication skills. In some children, the symptoms of speech understanding were observed. The rate of this improvement was slow but offered the autistic child better communication skills and social integration mainly with family members but also with our cochlear implant team and rehabilitation team. In communication the eye contact is also very important but this ability was the least improved in our group and as shown in literature.^5^

No increased hyperactivity associated with daily use cochlear implant was observed. The study showed that in autistic children the perception is very important for a child's sense of security and makes contact with parents easier, the contact that is limited by autism itself. Some parents pointed that with cochlear implant on the children are calmer.

## Conclusion

Autism is not a contraindication for cochlear implantation, but goals and expectations about the effects of auditory and language outcomes are different than in the group of children with profound hearing loss without any additional problems. Oral communication is not likely to be a realistic goal in those cases. The implantation results are also different for each child with autism.

Our study showed that the traditional methods of evaluating the results of cochlear implantation in children with autism are usually insufficient to fully assess the functional benefits. Evaluation to measure progress is challenging and any change in hospital fitting visit and rehabilitation routine may increase child's anxiety and influence the results. The implantation benefits should be assessed in a more comprehensive manner taking into account the limitations of communication resulting from the essence of autism. It is important that we share knowledge about these complex children with cochlear implants.

## Conflicts of interest

The authors declare no conflicts of interest.
